# The Overuse of Hepatobiliary Scintigraphy (HIDA) Scans: Are We Unnecessarily Using Nuclear Medicine Resources Instead of Proven Clinical Guidelines for Diagnosing Acute Cholecystitis?

**DOI:** 10.7759/cureus.73560

**Published:** 2024-11-12

**Authors:** Shelley Warner, Janavi Patel, Stacey L Tannenbaum, Rachel Kessler, Gary Lehr

**Affiliations:** 1 General Surgery, Broward Health Medical Center, Fort Lauderdale, USA; 2 College of Medicine, Nova Southeastern University Dr. Kiran C. Patel College of Osteopathic Medicine, Fort Lauderdale, USA

**Keywords:** cholecystitis, cholecystitis / diagnosis, gallbladder imaging modalities, gallstone disease, hida scan, tokyo guidelines

## Abstract

Background

Gallstone disease significantly burdens the United States healthcare system. While ultrasonography (US), physical exam, and laboratory findings are the recommended primary workup and diagnostic modalities, hepatobiliary scintigraphy (HIDA) scans are occasionally used as an adjunct for diagnosis. This study evaluates HIDA scan utilization in comparison to clinical and US findings based on the Tokyo guidelines for diagnosing acute cholecystitis.

Methods

This retrospective study included 159 patients admitted with gallbladder disease from January 1, 2019, to December 31, 2020. Patients were classified by the Tokyo guidelines as having no cholecystitis, suspected, or definite cholecystitis. The primary outcome was HIDA scan overutilization, defined as HIDA scans performed despite clinical criteria for cholecystitis. Secondary outcomes included HIDA scan use in complicated gallbladder disease and the effect of admission day on HIDA scan ordering.

Results

Of the 159 patients who underwent cholecystectomy, 101 (63.5%) met the Tokyo guidelines for suspected or definite cholecystitis. Over half, 54 (53.5%) of these patients received HIDA scans, indicating overutilization. Additionally, no significant difference in HIDA scan utilization was observed based on the day of admission. Among patients with complicated gallbladder disease, 29 (38.2%) underwent a HIDA scan, which was deemed unnecessary.

Conclusion

HIDA scans are significantly overutilized in patients meeting clinical criteria for cholecystitis based on the Tokyo guidelines and those with complicated gallbladder disease. Overuse increases healthcare costs and delays care. HIDA scans should be reserved for cases with inconclusive US results but high clinical suspicion for cholecystitis. Proper utilization and reduction of unnecessary HIDA scans could improve patient care efficiency and reduce healthcare expenditures.

## Introduction

Gallstone disease is a significant and costly issue within the healthcare system in the United States, leading to over 700,000 cholecystectomies and over a million hospitalizations annually [1]. Every year, about 15-20% of individuals with gallstones experience symptoms, making gallstone disease the leading cause of gastrointestinal-related inpatient hospital admissions [2,3]. There have been many advances in streamlining the diagnosis and operative intervention for cholecystitis to improve patient outcomes and hospital costs. The standard approach consists of a thorough history and physical examination, basic laboratory results, and imaging. There are a few options for diagnostic imaging, including gallbladder ultrasound (US), nuclear medicine hepatobiliary scintigraphy (generically referred to as HIDA), computed tomography (CT), and magnetic resonance imaging (MRI). Ordering patterns differ due to a variety of factors, including resource availability, patient presentation, and provider bias. Specifically, the indications for the use of HIDA scans described in the literature differ from what is observed in the clinical setting. 

The Tokyo guidelines are a validated tool to aid in the diagnosis and management of gallbladder disease. Specific diagnostic criteria for suspicion of and severity grading for cholecystitis are addressed in the most updated version of the Tokyo 2018 guidelines [4,5]. These criteria encompass physical examination findings of local signs of inflammation, including right upper quadrant abdominal pain or a positive Murphy’s sign; laboratory findings of systemic inflammation, including elevated temperature, elevated C-reactive protein, or elevated white blood cell count; and imaging findings indicative of acute cholecystitis, including gallbladder wall thickening, gallbladder distention, or pericholecystic fluid [5]. The Tokyo guidelines emphasize the necessity of imaging studies to confirm the diagnosis since no single physical examination finding or laboratory test can independently establish or exclude acute cholecystitis. The current recommendation is for ultrasonography use, but findings on other methods of imaging can be used to establish Tokyo criteria as well [5]. 

Although gallbladder US remains the primary diagnostic imaging modality for gallstones, there is a continued comparison with alternative diagnostic methods, specifically, HIDA scans [1,5,6]. The use of gallbladder US versus HIDA scan has been scrutinized in multiple studies comparing these tests’ sensitivity, accuracy, and utility [6-16]. While HIDA scans have superior diagnostic capabilities, the US remains the recommended primary modality for diagnosing acute cholecystitis due to it being a quick, effective, and affordable test [5,6]. The drawbacks of HIDA scans are due to the prolonged time to perform the study, the specialized team required, and additional costs. When discussing utilization of HIDA scans, it is suggested to have limited or selective use based on high-risk patients [9,13,14]. Additionally, when a patient presents with a complication of gallstone diseases such as choledocholithiasis, ascending cholangitis, or biliary pancreatitis, the standard of care is to perform a cholecystectomy during that index hospitalization, thus negating the need for further investigation of the cystic duct obstruction during a HIDA scan. This means there is usually no benefit to a HIDA scan in complicated biliary disease.

The sensitivity and specificity of the US when combined with clinical symptoms and laboratory values included in the Tokyo guidelines are found to be comparable to that of a HIDA scan [5]. A study conducted by Rodriguez et al. in 2016 corroborated these findings; when used in conjunction with clinical assessment and laboratory data, the US alone yields the same positive predictive value (PPV) for acute cholecystitis as a HIDA scan [9]. Another study conducted in 2013 that analyzed the necessity of a HIDA scan for the diagnosis of cholecystitis found that the US in isolation demonstrated a PPV of 96% for diagnosing cholecystitis, which is consistent with the 96% PPV observed for HIDA scans [14]. While the sensitivity and specificity rates of the US have significant variability reported, with some as low as 80-85% due to operator dependence, these US findings, when coupled with clinical evaluation and laboratory data, can still produce identical clinical diagnoses when compared to HIDA scans [5]. The use of HIDA scans, while diagnostically more accurate, may increase costs and delay patient care [5]. Moreover, the postponement of a diagnosis associated with the use of HIDA scans can elevate the risk of adverse patient outcomes [10,17].

This study aims to assess the proper use of HIDA scans based on the Tokyo guidelines because the disadvantages of HIDA scans have numerous impacts on patients and the health care system. A HIDA scan itself is three times as expensive as an ultrasound, and patients incur a cost of up to $1600 extra [9,13]. In addition, patients also have increased lengths of stay when they undergo HIDA scans [14]. One potential confounding factor to ordering HIDA scans is the particular day of the week of admission. Because non-urgent/emergent cholecystectomies are not routinely performed on the weekend at our institution, we hypothesized that more patients would undergo HIDA scans when admitted on Friday/Saturday, as compared to Sunday. Overall, our hypothesis is that HIDA scans are overutilized in clinical practice, as they are ordered even when sufficient evidence exists for a patient to undergo a cholecystectomy, rendering HIDA scans redundant, costly, and unnecessary imaging modalities in the workup of acute cholecystitis.

## Materials and methods

This was a retrospective cohort study of patients admitted with gallbladder disease to Broward Health Medical Center, Fort Lauderdale, United States (n=159). The Institutional Review Board of the hospital provided an exempt status for this study due to the retrospective nature of the study and minimal risk to study subjects. Inclusion criteria consisted of patients who were 18 years and older admitted to the hospital from January 1, 2019, to December 31, 2020, who received gallbladder US or CT scan with or without HIDA scan. Patients were excluded if they did not undergo preoperative imaging during the same hospital admission as the cholecystectomy. The primary outcome was to determine if HIDA scans were overutilized in the community-based hospital. Overutilization was defined as patients who underwent HIDA scans despite meeting clinical criteria for cholecystitis based on the Tokyo guidelines. The secondary outcomes were the use of HIDA scans in complicated gallbladder disease and to determine if the day of admission affected HIDA scan ordering practices. The complications of gallbladder disease were defined as patients who presented with choledocholithiasis and/or gallstone pancreatitis. Concern for choledocholithiasis was defined as ductal dilation read by a radiologist on a US or CT scan (>7 mm) and an elevated bilirubin (>1.5 mg/dL). Gallstone pancreatitis was defined as an elevated lipase (>80 U/L). 

The Tokyo guidelines were used to classify patients into criteria based on physical examination and laboratory and imaging findings. The three categories were criteria not met (no cholecystitis), suspected, and definite cholecystitis. These three groups were then used to evaluate the percentage of HIDA scans performed based on diagnostic criteria. HIDA scan results and gallbladder pathology were also evaluated for patients in each category. HIDA results were categorized as normal, delayed, non-visualization of the gallbladder, or radiotracer in the liver without visualization of the gallbladder or bowel. Gallbladder pathology was grouped as acute, gangrenous, or chronic cholecystitis. Demographic information for each patient was recorded from the electronic medical record (EMR), including age (years), sex (male/female), race (Black, White, Asian), ethnicity (Hispanic/non-Hispanic), as well as comorbidities that increase the risk for gallstones, including body mass index (BMI) (kg/m^2^), and a diagnosis of diabetes mellitus (yes/no). Other variables collected were the day of the week on admission either a weekday (Monday-Thursday), Friday to Saturday, or Sunday to determine if the number of HIDA scans ordered differed by the day of admission.

Statistical analysis

Demographic data and other characteristics of patients are displayed as means and standard deviations (SD) for numeric data. Categorical data are expressed as frequencies and percentages. Chi-square or Fisher’s exact test (as applicable) was used to compare associations of categorical variables to one another. Chi-square tests were performed, evaluating the patient’s severity of cholecystitis based on Tokyo guidelines and variables including HIDA scans performed and the day of the week of admission. The correlation of gallbladder pathology and HIDA scan results and the Tokyo guideline classification was measured for strength and association with Kendall's tau-b (τb) correlation coefficient. A significance level of 0.05 was used for all tests. Data analysis was performed using IBM SPSS Statistics for Windows, Version 29 (Released 2023; IBM Corp., Armonk, New York, United States).

## Results

A total of 259 patients were admitted to the one community hospital over two years and received a gallbladder US or CT scan with or without a HIDA scan. All US and CT scans were performed prior to the HIDA scans. Included in this study were 159 (61.4%) patients who subsequently underwent cholecystectomy. Three patients did not receive a US and had a CT scan as the initial imaging. Demographic characteristics are displayed in Table 1. The mean age of the total sample was 52.9 years (SD 18.4) with a mean BMI of 30.5 kg/m^2^ (SD 7.4). The majority of the sample were female (60.0%), White (70.0%), non-Hispanic (77.0%), and did not have diabetes (84%). When comparing those patients who received HIDA scans versus those who did not, no significant demographic differences were seen between the two groups (Table 1).

**Table 1:** Demographic data for all patients and between cohorts based on imaging modality *T statistics with p-value based on two-sided t-test with unequal variances; ^+^Pearson chi-squared statistic with p-value based on chi-square test BMI: body mass index; HIDA scan: hepatobiliary scintigraphy

Demographic	All Patients (n=159)	HIDA (n=75)	No HIDA (n=84)	Test Statistic	p-value
Age, mean (SD)	52.9 (18.4)	52.2 (17.5)	53.9 (19.2)	1.975*	0.68
BMI, mean (SD)	30.5 (7.4)	30.6 (8.2)	30.2 (6.9)	1.977*	0.738
Sex (%)				1.841^+^	0.175
Male	64 (40%)	26 (35%)	38 (45%)	-	-
Female	95 (60%)	49 (65%)	46 (55%)	-	-
Race (%)				0.719^+^	0.702
Black	44 (28%)	22 (29%)	22 (26%)	-	-
White	112 (70%)	51 (68%)	61 (73%)	-	-
Asian	3 (2%)	2 (3%)	1 (1%)	-	-
Ethnicity (%)				0.029^+^	0.865
Hispanic	37 (23%)	17 (23%)	20 (24%)	-	-
Non-Hispanic	122 (77%)	58 (77%)	64 (76%)	-	-
Diabetes (%)				0.020^+^	0.887
Yes	24 (15%)	11 (15%)	13 (15%)	-	-
No	135 (84%)	64 (85%)	71 (85%)	-	-

**Figure 1:**
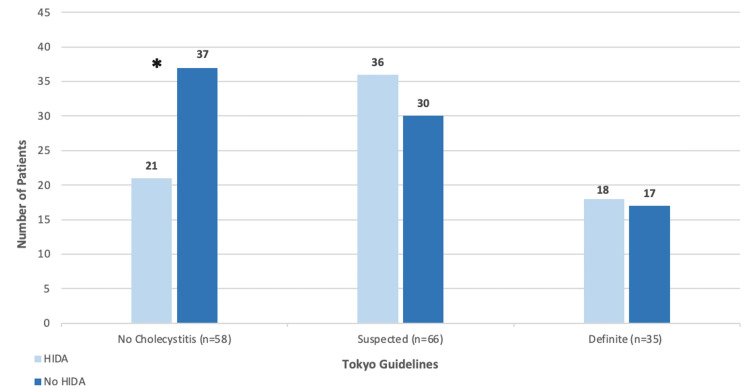
Tokyo guidelines classification and count of HIDA scans performed *Significant difference between two groups with a p-value of 0.036 HIDA scan: hepatobiliary scintigraphy

Of the 159 patients who underwent cholecystectomy, 101 met the Tokyo guidelines for suspected or definite cholecystitis (Figure 1). There was no significant difference in the number of scans performed for the suspected and definite groups. Notably, 54 (53.5%) total patients who met suspected or definite criteria for cholecystitis based on the Tokyo guidelines underwent a HIDA scan in addition to the gallbladder ultrasound (Figure 1). There were significantly fewer HIDA scans performed (yes=21; no=37; p=0.036) for the “no cholecystitis” group based on Tokyo guidelines.

Regarding outcomes of the HIDA scans, 16 (21.3%) were normal, six (8.0%) showed delayed visualization of the gallbladder, 47 (62.7%) showed non-visualization of the gallbladder, and four (5.3%) showed radiotracer in the liver only (Table 2). Of the 16 patients with normal HIDA scans, 11 (68.8%) met the criteria of suspected or definitive cholecystitis. There was no direct correlation between HIDA scan results and Tokyo guideline classification (τb=0.059, p=0.576). A closer examination of gallbladder pathology demonstrated acute cholecystitis in 91 (57.2%) patients (Table 3). Of these 91 patients, 40 (44.0%) met the criteria for suspected and 21 (23.1%) for definite cholecystitis based on Tokyo guidelines. For chronic cholecystitis, only five (10.0%) of the 50 patients met the criteria for definite cholecystitis, 22 (44.0%) patients met the criteria for suspected, and 23 (46.0%) patients were categorized as having no acute cholecystitis based on Tokyo guidelines. Of the 58 patients who had a cholecystectomy without meeting the criteria for cholecystitis, 23 (40.0%) had chronic cholecystitis on their pathology (Table 3). 

**Table 2:** HIDA scan results based on Tokyo guideline classification ^+ ^: Τ_b _Kendall Tau B’s correlation coefficient between gallbladder pathology and cholecystitis classification; * : p-value based on Kendall Tau b association of variables HIDA scan: hepatobiliary scintigraphy

HIDA Result	Total (n=75)	No Cholecystitis (n=21)	Suspected (n=36)	Definite (n=18)	Τ_b_^+^	p-value
Normal	16	5	9	2	0.058	0.576*
Delayed	6	1	4	1		
Non-visualization	47	13	20	14		
Radiotracer in Liver	4	2	2	0		
Other	2	0	1	1		

**Table 3:** Gallbladder pathology based on Tokyo guidelines ^+ ^: Τ_b_ Kendall Tau B’s correlation coefficient between gallbladder pathology and cholecystitis classification; * : p-value based on Kendall Tau b association of variables

Gallbladder Pathology	Total (n=159)	No Cholecystitis (n=58)	Suspected (n=66)	Definite (n=35)	Τ_b_^+^	p-value
Acute (%)	91	30 (33)	40 (44)	21 (23)	-0.11	0.130*
Gangrenous (%)	18	5 (28)	4 (22)	9 (50)		
Chronic (%)	50	23 (46)	22 (44)	5 (10)		

The day of admission was grouped into three categories: weekday, Friday/Saturday, and Sunday. Most admissions were during the weekday, which included only Monday through Thursday, with 94 (59%) patients (Table 4). Of those 94 patients, 45 (47.9%) underwent a HIDA scan, compared to 49 (52%) of patients who did not undergo a HIDA scan. A total of 41 (26%) patients were admitted on Friday or Saturday, with 22 (53.7%) receiving HIDA scans and 19 (46%) who did not. Lastly, the Sunday admission total was 24 (15%) with eight (33.3%) patients receiving HIDA scans and 16 (66%) who did not. There were no significant differences between the proportion of patients who received a HIDA scan compared to no HIDA scan between the three groups of weekday, Friday/Saturday, and Sunday admission (chi-square 2.55, p=0.279) (Table 4). 

**Table 4:** HIDA performed based on the day of the week admission * : Pearson chi-squared statistic with p-value based on chi-square test HIDA scan: hepatobiliary scintigraphy

Admission Day of Week	Total (n=159, %)	No HIDA (n=84)	Yes HIDA (n=75)	Test Statistic	p-value
Weekday (%)	94	49 (52)	45 (48)	2.55^*^	0.279
Friday/Saturday (%)	41	19 (46)	22 (54)		
Sunday (%)	24	16 (67)	8 (33)		

**Figure 2:**
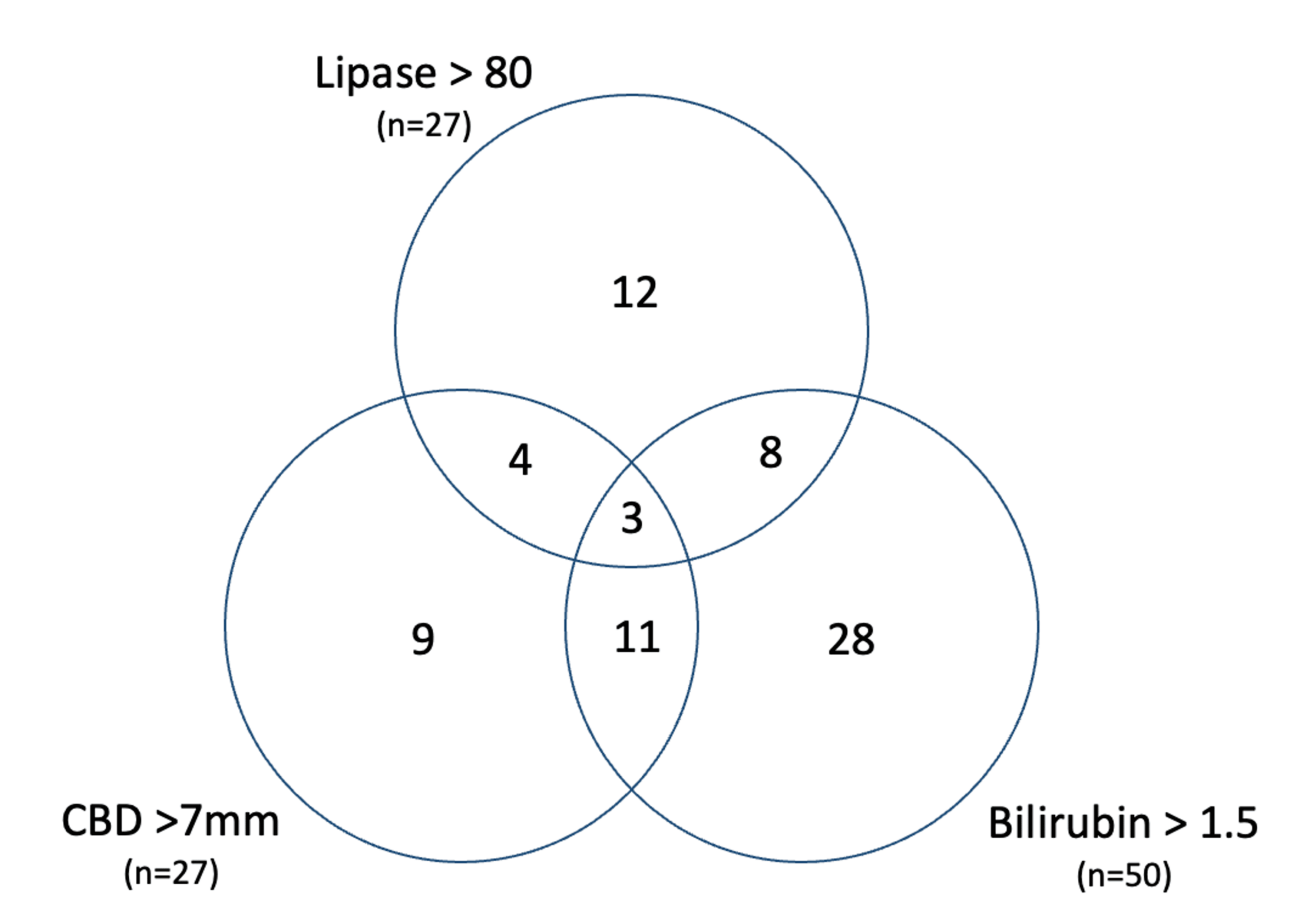
Venn diagram depicting the breakdown of findings indicating complications of gallbladder disease CBD: common bile duct

The Venn diagram (Figure 2) portrays how the complications of gallbladder disease overlap between patients. These include dilated common bile duct (CBD) of greater than 7 mm, elevated bilirubin of >1.5 mg/dL, and elevated lipase of >80 U/L. The intersection of CBD dilation and bilirubin was the greatest with 14 patients. Bilirubin levels were elevated in 50 patients, lipase was elevated in 27 patients, and CBD dilation in 27 patients. All three markers of gallstone complications were evident in only three patients. The percentage of patients who received a HIDA scan and had at least one of the complications of gallbladder disease is presented in Table 5. There were a total of 76 unique patients who presented with at least one finding of complicated gallbladder disease. There was a significant difference between patients who had a HIDA scan compared to those who did not when looking at patients with elevated bilirubin (P=0.003). There were no significant differences between HIDA scans performed in CBD dilation and elevated lipase groups (p=0.357, p=0.756, respectively) (Table 5). Of the 76 patients who presented with possible choledocholithiasis or gallstone pancreatitis, 29 (38.2%) of those patients also underwent an HIDA scan. When comparing complicated gallbladder disease to uncomplicated disease, there were significantly (p=0.029) fewer patients who received a HIDA scan when they had complicated gallbladder disease (Table 5).

**Table 5:** Breakdown of patients with complications of gallbladder disease and crosstab of complications vs. no complications with HIDA scans ^+ ^: Z value; * : Pearson chi-squared statistic with p-value based on chi-square test HIDA scan: hepatobiliary scintigraphy; CBD: common bile duct; elevated bilirubin >1.5; elevated lipase >80

Complications	All Patients (n=159)	HIDA (n=75)	No HIDA (n=84)	Test Statistic	p-value
Dilated CBD (%)	27	10 (37)	17 (63)	-1.15^+^	0.246
Elevated bilirubin (%)	50	15 (30)	35 (70)	-2.9^+^	0.003
Elevated lipase (%)	27	12 (44)	15 (56)	-0.31^+^	0.756
Total				4.74*	0.029
With complications (%)	76	29 (38)	47 (62)		
Without complications (%)	83	46 (55)	37 (45)		

## Discussion

Our study evaluates the utilization of HIDA scans, specifically the excessive use of this imaging modality for gallbladder disease diagnosis. We found that despite initial ultrasound, physical examination, and laboratory results demonstrating high suspicion for cholecystitis, patients were subsequently subjected to further imaging with a HIDA scan. Additionally, there were many patients with complicated diseases who underwent HIDA scans, which were never indicated. Our study was unique in that we evaluated the appropriateness of HIDA scan utilization based on each patient’s Tokyo guidelines categorization. While we recognize that HIDA scans demonstrate superior sensitivity and accuracy compared to the US alone, their benefit in diagnosis is only valuable when the initial workup, including physical exam, laboratory values, and US findings, are inconclusive. HIDA scans should be reserved for only specific cases in which the diagnosis is unclear [15,16]. 

Our analysis revealed significant redundant imaging for our patients. Of the 159 patients who underwent cholecystectomy, 101 were grouped into the suspected or definite cholecystitis groups based on the Tokyo guidelines. Of these patients, 54 (53.5%) underwent a HIDA scan, with 18 (51.4%) of the definite group receiving a HIDA scan. Additionally, of the 75 (48.7%) patients in our study who underwent HIDA scans, 54 (72.0%) met the Tokyo guidelines for suspected or definite cholecystitis, with 18 (46.1%) of the definite group undergoing HIDA scans. There is no significant difference in the number of HIDA scans performed versus those not performed in the suspected and definite groups. This indicates that HIDA scans were ordered unnecessarily, without evaluation of the likelihood of acute cholecystitis. Most of these patients would likely have undergone a cholecystectomy regardless of HIDA scan results due to the findings from their US or CT, laboratory testing, and physical examination findings. In fact, 16 (21.3%) patients had normal HIDA scans and still underwent a cholecystectomy, indicating the results of the HIDA had little impact on surgical decision-making.

There were significantly fewer HIDA scans ordered in the subset of patients categorized as “no cholecystitis” (n=58) based on the Tokyo guidelines classification. This is most likely attributed to the inclusion of patients without cholecystitis but who presented with complications of gallbladder disease, including choledocholithiasis and/or biliary pancreatitis. Patients with complications of gallbladder disease may not always present with the same imaging findings or symptoms as those with acute cholecystitis; however, these patients are still recommended to undergo cholecystectomy during their initial hospital admission and thus are included in our cohort of patients. This explains the reason why fewer HIDA scans were performed in the no cholecystitis group.

The Tokyo guidelines are specifically designed for evaluating acute cholecystitis. In our patient population, 91 patients had pathology-confirmed acute cholecystitis. The majority, 61 (67%) of these patients, appropriately met the Tokyo guidelines criteria for suspected or definite cholecystitis. Many (n=50) patients had pathology-confirmed chronic cholecystitis. Overall, 58 (46%) patients did not meet the criteria for cholecystitis based on the Tokyo guidelines but were found to have acute, gangrenous, or chronic cholecystitis on pathology. The overall sensitivity of the US to detect chronic cholecystitis is only about half when compared to detecting acute cholecystitis [17]. This may be in part due to the US being less likely to detect wall thickening in chronic cholecystitis or gangrenous cholecystitis [5,17]. Patients with chronic or gangrenous cholecystitis are more likely to have equivocal ultrasound findings despite their symptoms. This subset of patients would benefit from a HIDA scan, as they would present with high clinical suspicion of cholecystitis but equivocal ultrasound findings [5,6]. Our results do not demonstrate a different rate of HIDA scan use between the pathologic findings of acute, chronic, or gangrenous cholecystitis. This again emphasizes the probable overuse of HIDA scans without patient-specific selection when ordering. Based on our findings, we recommend that HIDA scans be reserved for patients who present with a high clinical suspicion for cholecystitis and complete an initial nondiagnostic gallbladder US. 

In our study, there was clear overuse of HIDA scans in the population of complicated gallbladder disease. A total of 76 patients presented with complications of gallbladder disease, and 29 (39%) patients still underwent a HIDA scan. All of these patients should have undergone cholecystectomy based on initial laboratory and imaging findings, regardless of an acute cholecystitis diagnosis. There was a statistically significant larger number of patients who did not undergo a HIDA scan when the bilirubin was elevated (p=0.003). A likely explanation for the limited use of HIDA scans in this situation is that these patients underwent alternative diagnostic imaging, such as MRIs, and thus providers may have determined that a HIDA scan was not essential. When the patients had biliary pancreatitis evidenced by an elevated serum lipase level, they were just as likely to undergo a HIDA scan versus no HIDA scan. Overall, 39% of patients with complicated gallbladder disease still underwent a HIDA scan, and this number should have been approaching zero. We hypothesize that these findings are due to the community-based hospital setting in which the majority of patients are admitted to a medical service with a complete workup and diagnostic imaging ordered prior to consultation by the general surgery service. 

In our community hospital, the weekends are usually reserved for urgent and emergent cases, and therefore, cholecystitis patients may wait upwards of 48 hours prior to surgical intervention. We predicted these patients would be more likely to have a HIDA scan completed when waiting over the weekend; however, we found that there was no difference between the number of HIDAs performed when patients were admitted on Friday/Saturday, Sunday, or a weekday. This finding suggests that the ordering of HIDA scans is consistent across different days of the week, indicating no significant variation in clinical decision-making based on the day of the encounter. Therefore, there is no bearing on which day of the week HIDA scans are ordered or performed and overutilized.

The increased time and cost to the healthcare system must be considered when ordering HIDA scans. Previous data demonstrated that patients who received HIDA scans had a significant delay in surgical intervention by almost two days compared to patients who only had a US [9]. Delaying surgery for acute cholecystitis can also lead to increased morbidity and mortality from operative intervention, including bile duct leaks or major bile duct injuries, which increases healthcare costs by over $10,000 [18]. Moreover, the cost of performing a HIDA scan is three times more expensive than an ultrasound [14]. The added length of stay and increased risk of complications with delayed cholecystectomy pose significant expense to the patient and hospital system, which can result from the unnecessary ordering of a HIDA scan [14]. Therefore, the decision to order HIDA scans should be made with careful consideration, reserved for cases where the results would significantly alter the plan of care for the patient. 

In our patient population, the majority (61.9%) of patients who did not meet Tokyo guidelines for cholecystitis and then underwent a HIDA scan were found to have non-visualization of their gallbladder. This reinforces the idea that when there is a high suspicion for cholecystitis but equivocal initial findings with laboratory values and ultrasound findings, a HIDA scan, with increased sensitivity and specificity, can provide meaningful information to help establish the diagnosis. This illustrates the proper utilization of HIDA scans; that is, the result of the scan changed the hospital course of these patients and resulted in the appropriate surgical management with the performance of a cholecystectomy.

There are a few limitations to this study. This was a single-center cohort with a multitude of different providers conducting patient workups. The decreased standardization of care seen in a community setting like this hospital-based study may be a reason for the overuse of HIDA scans seen in our study. Increasing the sample and using data from other hospitals and providers may help to overcome this problem. Additionally, multi-center data from both community and academic settings will help increase the generalizability of HIDA scan overuse. One strength of this study is that the majority of hospitals in the United States. are non-university community facilities; these outcomes are more likely to be relevant to a large part of the United States healthcare system.

Comparative effectiveness research, optimization of diagnostic protocols, investigation of long-term patient outcomes, and cost-effectiveness analyses are essential in managing patients with cholecystitis, as it is one of the leading causes of inpatient admission in the United States. This includes exploring new imaging technologies and refining existing modalities to enhance diagnostic accuracy. Clarifying protocols to appropriately employ HIDA scans or other diagnostic imaging is imperative to reduce treatment delays and minimize healthcare costs. Moreover, the integration of artificial intelligence and machine learning in imaging interpretation could improve the diagnostic speed and accuracy of USs, which may eliminate the need for HIDA scans in cholecystitis workups in the future. Large-scale, multicenter trials comparing patient outcomes based on different diagnostic pathways could offer more definitive clinical guidelines.

## Conclusions

While HIDA scans offer excellent diagnostic evidence of acute cholecystitis, they are costly and time-consuming and should only be used in equivocal cases. Our study demonstrated that they are often overutilized in patients who can be diagnosed based on Tokyo guidelines with ultrasound findings in conjunction with physical exam and laboratory findings. Additionally, we found HIDA scans are being used in patients with complicated gallstone disease, which is not an indication for HIDA scans. Ordering a HIDA scan must be judiciously balanced against the costs, logistical challenges, and potential treatment delays. US remains a practical, effective, and efficient primary diagnostic tool for establishing a diagnosis of acute cholecystitis, especially when combined with the clinical assessment of the patient and interpretation of their laboratory tests.

## References

[1] Bar-Meir S (2021). Gallstones: prevalence, diagnosis and treatment. Isr Med Assoc J.

[2] Shaffer EA (2006). Gallstone disease: epidemiology of gallbladder stone disease. Best Pract Res Clin Gastroenterol.

[3] Kimura Y, Takada T, Kawarada Y (2007). Definitions, pathophysiology, and epidemiology of acute cholangitis and cholecystitis: Tokyo guidelines. J Hepatobiliary Pancreat Surg.

[4] Okamoto K, Suzuki K, Takada T (2018). Tokyo guidelines 2018: flowchart for the management of acute cholecystitis. J Hepatobiliary Pancreat Sci.

[5] Yokoe M, Hata J, Takada T (2018). Tokyo guidelines 2018: diagnostic criteria and severity grading of acute cholecystitis (with videos). J Hepatobiliary Pancreat Sci.

[6] Kiewiet JJ, Leeuwenburgh MM, Bipat S, Bossuyt PM, Stoker J, Boermeester MA (2012). A systematic review and meta-analysis of diagnostic performance of imaging in acute cholecystitis. Radiology.

[7] Kalimi R, Gecelter GR, Caplin D (2001). Diagnosis of acute cholecystitis: sensitivity of sonography, cholescintigraphy, and combined sonography-cholescintigraphy. J Am Coll Surg.

[8] Alobaidi M, Gupta R, Jafri SZ, Fink-Bennet DM (2004). Current trends in imaging evaluation of acute cholecystitis. Emerg Radiol.

[9] Rodriguez LE, Santaliz-Ruiz LE, De La Torre-Bisot G (2016). Clinical implications of hepatobiliary scintigraphy and ultrasound in the diagnosis of acute cholecystitis. Int J Surg.

[10] Gurusamy KS, Davidson C, Gluud C, Davidson BR (2013). Early versus delayed laparoscopic cholecystectomy for people with acute cholecystitis. Cochrane Database Syst Rev.

[11] Kaoutzanis C, Davies E, Leichtle SW (2015). Is hepato-imino diacetic acid scan a better imaging modality than abdominal ultrasound for diagnosing acute cholecystitis?. Am J Surg.

[12] Lambie H, Cook AM, Scarsbrook AF, Lodge JP, Robinson PJ, Chowdhury FU (2011). Tc99m-hepatobiliary iminodiacetic acid (HIDA) scintigraphy in clinical practice. Clin Radiol.

[13] Johnson H Jr, Cooper B (1995). The value of HIDA scans in the initial evaluation of patients for cholecystitis. J Natl Med Assoc.

[14] Bernescu I, Eng OS, Potdevin L, Monteiro R, Mino J, Chang EI, Davidov T (2013). Is HIDA scan necessary for sonographically suspicious cholecystitis?. J Curr Surg.

[15] Kaoutzanis C, Davies E, Leichtle SW, Welch KB, Winter S, Lampman RM, Arneson W (2014). Abdominal ultrasound versus hepato-imino diacetic acid scan in diagnosing acute cholecystitis--what is the real benefit?. J Surg Res.

[16] Gupta A, Osher M, Pallas A, Wang X (2014). Comparison of hepatobiliary iminodiacetic acid (HIDA) scan, ultrasound (US), computer tomography (CT) in evaluation of acute cholecystitis. J Nucl Med.

[17] Room H, Wood A, Ji C, Nightingale H, Toh S (2022). Performance of ultrasound in the diagnosis of cholecystitis: not so (ultra)sound?. Ann R Coll Surg Engl.

[18] de Mestral C, Hoch JS, Laupacis A, Wijeysundera HC, Rotstein OD, Alali AS, Nathens AB (2016). Early cholecystectomy for acute cholecystitis offers the best outcomes at the least cost: a model-based cost-utility analysis. J Am Coll Surg.

